# A Multi-Sensor Fusion Underwater Localization Method Based on Unscented Kalman Filter on Manifolds

**DOI:** 10.3390/s24196299

**Published:** 2024-09-29

**Authors:** Yang Wang, Chenxi Xie, Yinfeng Liu, Jialin Zhu, Jixing Qin

**Affiliations:** 1 Department of Automation, Beijing Information Science and Technology University, Beijing 102206, China; xiechenxi@bistu.edu.cn (C.X.); jlzhu@bistu.edu.cn (J.Z.); 2 Department of Applied Science, Beijing Information Science and Technology University, Beijing 102206, China; dxjm_lyf@163.com; 3 State Key Laboratory of Acoustics, Institute of Acoustics, Chinese Academy of Sciences, Beijing 100190, China; qjx@mail.ioa.ac.cn

**Keywords:** unscented Kalman filter, underwater localization, manifolds, Lie groups

## Abstract

In recent years, the simplified computation of position and velocity changes in nonlinear systems using Lie groups and Lie algebra has been widely used in the study of robot localization systems. The unscented Kalman filter (UKF) can effectively deal with nonlinear systems through the unscented transformation, and in order to more accurately describe the robot localization system, the UKF method based on Lie groups has been studied successively. The computational complexity of the UKF on Lie groups is high, and in order to simplify its computation, the Lie groups are applied to the manifold, which efficiently handles the state and uncertainty and ensures that the system maintains the geometric constraints and computational simplicity during the updating process. In this paper, a multi-sensor fusion localization method based on an unscented Kalman filter on manifolds (UKF-M) is investigated. Firstly, a system model and a multi-sensor model are established based on an Autonomous Underwater Vehicle (AUV), and a corresponding UKF-M is designed for the system. Secondly, the multi-sensor fusion method is designed, and the fusion method is applied to the UKF-M. Finally, the proposed method is validated using an underwater cave dataset. The experiments demonstrate that the proposed method is suitable for underwater environments and can significantly correct the cumulative error in the trajectory estimation to achieve accurate underwater localization.

## 1. Introduction

When an Autonomous Underwater Vehicle (AUV) performs complex oceanographic tasks such as underwater exploration, seafloor topographic mapping, and environmental monitoring, the localization and navigation of the AUV becomes a key issue due to the special characteristics of the underwater environment, including signal attenuation, noise interference, and dynamic changes, which make the use of conventional satellite navigation systems impossible. To provide accurate real-time position information to the navigation system, AUVs are typically equipped with a variety of sensors, such as an inertial measurement unit (IMU), Doppler velocity log (DVL), and depth sensor. These sensors provide detailed information about the speed, direction, and depth of the AUV while operating in the underwater environment, and the accuracy of underwater localization can be improved by realizing the synergy between the sensors for the position estimation of the AUV [1,2].

The accuracy of AUV 3D attitude estimation is of critical importance during underwater navigation. Filtering algorithms are commonly employed for robot attitude estimation, with traditional filtering methods utilizing Bayesian estimation such as the Kalman filter (KF) frequently encountering difficulties, particularly when confronted with nonlinear dynamic systems [3]. These challenges have led to the development of more sophisticated filtering methods, including the extended Kalman filter (EKF) [4] and the unscented Kalman filter (UKF) [5]. These methods exhibit enhanced accuracy and robustness in dealing with nonlinear estimation tasks.

Underwater environments are characterized by complex topographies, such as submarine mountains, canyons, coral reefs, etc., which usually exhibit nonlinear three-dimensional structures. An AUV needs to deal with these complex underwater topographies and environments when equipped with multiple sensors for underwater navigation and localization. Manifolds offer a natural framework for processing and simplifying high-dimensional nonlinear data. The fusion of multi-sensor information using nonlinear Manifolds [6,7] can improve the accuracy and reliability of the AUV’s 3D attitude estimation, thereby enhancing the precision of underwater navigation and localization [8,9]. The Lie group, as a manifold with a group structure, is better suited to handle rotations and transformations in nonlinear state spaces when filtered using the geometric constraints inherent in its own elements. This avoids mathematical problems such as singularity and linearization errors [10,11]. In recent years, Lie groups have been widely used in robot navigation. For mobile robots, it is necessary to represent the robot’s pose and position relative to the environment in a 3D space. Using the Special Orthogonal Group SO(3) in Lie groups to represent rotations can avoid the singularity problem of Euler angles and the redundancy of quaternions. Meanwhile, the Special Euclidean group SE(3) combines the representation of translations and rotations and is able to completely describe the robot’s position in space [12]. Relying on Kalman filtering in Lie groups, Fernandes [13] et al. investigated an extended Kalman filter built into a smoother mapping that aggregates the position, velocity, attitude, and IMU deviations of a UAV in a single element. Experiments are conducted to test the superiority of a loosely coupled GNSS/INS integrated navigation scheme utilizing Lie groups over multiplicative quaternions and Euler-based navigation schemes. Therefore, the state of a robot during its movement usually includes its position, velocity, acceleration, and attitude, and the representation of the position and attitude relies on the Lie group. By estimating the Lie group, the discontinuity and ambiguity of dealing with angles directly in a Euclidean space can be avoided.

To improve the accuracy of AUV position estimation, combining Lie groups with filters has become a major research direction [14]. In recent years, there has been a great deal of interest in the application of the invariant extended Kalman filter (IEKF) [15,16] and the unscented Kalman filter (UKF) [17,18] on Lie groups. Du, Siyuan, et al. [19] proposed a nonlinear strapdown inertial navigation system (SINS)/global navigation satellite system (GNSS) combined navigation estimation algorithm based on a Lie group manifold space. The nonlinear algorithm used is based on the unscented Kalman filter, and the proposed algorithm is experimentally demonstrated to have higher accuracy and good consistency compared with the existing combined navigation algorithms. Jeong, et al. [20] proposed a sensor fusion method for unmanned underwater vehicle navigation by combining Lie groups with extended Kalman filters, and experimentally demonstrated that the EKF on Lie groups can be accurately discretized in a differentiable space, which improves the accuracy of navigation. Therefore, the filters on Lie groups have been demonstrated to maintain consistency and stability in systems to a greater extent than filters applied on conventional Euclidean spaces. Furthermore, the geometric and algebraic structure of Lie groups has been utilized to enhance the accuracy and robustness of robot state estimation in nonlinear estimation.

The invariant extended Kalman filter (IEKF) leverages the inherent symmetry of the dynamical system to optimize the filtering performance. When the dynamics and observation model of the system are invariant under the action of Lie groups, the IEKF provides numerical stability and improved performance by maintaining this invariance [21,22]. Ross Hartley et al. [23] designed a contact-assisted inertial navigation observer for a 3D bipedal robot based on the IEKF, and demonstrated that the IEKF provides better accuracy in state estimation. Easton R. Potokar et al. [24] demonstrated that the IEKF can be applied to underwater navigation and that it is capable of faster convergence in terms of long-term localization when navigation is performed underwater by fusing the sensor information from the IMU and DVL. The IEKF can thus be applied to AUV dynamical systems with inherent symmetry, significantly reducing the estimation error and improving the accuracy of the filtering by naturally representing the system over the SE(3) group.

The unscented Kalman filter, as an effective nonlinear filter, circumvents the linearization error present in the extended Kalman filter by employing Sigma points, which are representative points selected around the estimated mean to approximate the true probability distribution [25]. Brossard et al. [26] derived simple left- and right-variant unscented Kalman filters on Lie groups and applied them to robot localization. The experiments demonstrated that this improved method outperforms the traditional UKF. However, the computational complexity of the UKF is higher than that of the EKF, and the computational and storage requirements are significantly higher for systems of a higher dimensionality [27]. Brossard et al. [28] simplified the computation and extended the unscented Kalman filters on the Lie group to generalize them to all manifolds. Meanwhile, corresponding localization frameworks were designed for different application scenarios of robots. The Lie group possesses both group and manifold structures. Its group operation is smooth under the manifold structure, and local linearization through Lie algebra enhances the computational efficiency and suitability for real-time systems.

In this paper, a multi-sensor fusion method based on the unscented Kalman filter on manifolds for underwater navigation is studied using the unscented Kalman filter framework of [28]. The main contributions of this paper are as follows:

1. Accurate AUV kinematic state modeling in an $SE_{2}(3)$ space and sensor modeling for the hydroacoustic sensors of the IMU, DVL, depth sensor, and gyroscope are established;
2. The retraction and inverse retraction functions are established for this AUV system to realize the propagation of the Sigma points of the Lie algebra state uncertainty between the Lie group space and the Li algebra space, and two different propagation methods, left-equivariant and right-equivariant, are designed based on this;
3. A manifolds-based UKF algorithm in this $SE_{2}(3)$ space is constructed to estimate and update the state of the AUV. The feasibility of the proposed algorithm as well as its implementation are finally verified using a real underwater cave dataset to ensure the improvement of the localization accuracy of the AUV.

The remainder of this paper is organized as follows: Section 2 presents the system model, including the motion model of the AUV, the sensor models of the IMU, DVL, depth sensor, and magnetometer. The specifics and intricate workings of the proposed algorithm are elaborated upon in Section 3. The experimental results based on AUV underwater cave data are analyzed in Section 4. Finally, Section 5 summarizes the principal conclusion of this work and discusses future work.

## 2. Establishment of System Model

### 2.1. AUV Model Description

This system focuses on the efficient localization method of AUV in an underwater environment that does not have a flat terrain, when the motion of the AUV can be regarded as an autonomous motion of rotation and translation on a three-dimensional nonlinear manifold. The Lie group, as a mathematical structure that is both a group and a smooth manifold, can be used to represent rotations and translations in a three-dimensional space through the Special Euclidean Group SE(3), which is used to characterize the robot’s pose and position.

**Figure 1:**
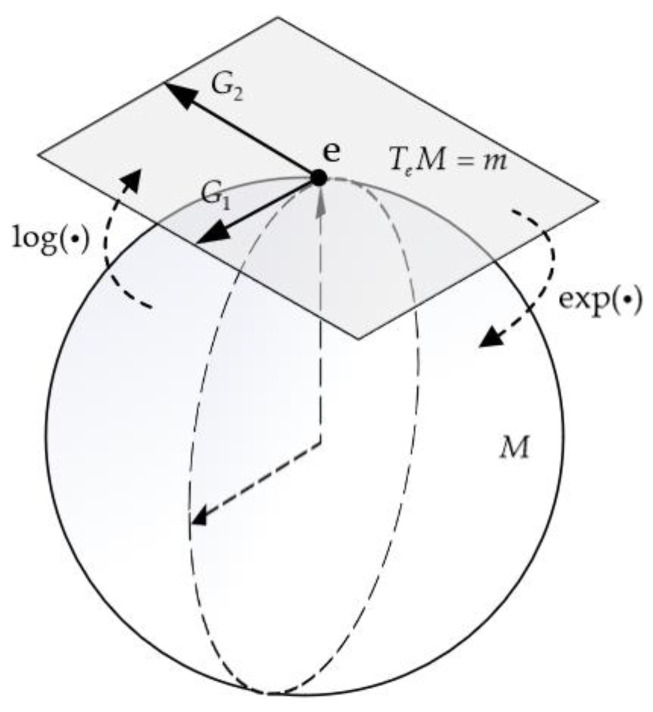
The Transformation of Lie Groups and Lie algebras.

Since Lie groups are inherently nonlinear, a Lie algebra is a local linearization of a Lie group, i.e., a Lie group’s tangent space in the neighborhood of the unit element. As in Figure 1, $T_{e}M$ denotes the tangent space of the Lie group manifold M at point *e*, which describes the “local linear approximation” of the point, and $G_{1}$ and $G_{2}$ define two basis vectors that provide the base for the vectors in the tangent space. The transition from Lie groups to Lie algebras transforms the AUV from a local motion of the surface to a linear motion. Elements of Lie algebra can be mapped to elements of the corresponding Lie group by the exponential map exp(·), and points on the Lie group can be mapped back to the tangent space by logarithmic mapping log(·). The exponential map exp(·) is used to generate complete rotations and translations from small changes, and elements in the Lie algebra can be viewed as infinitesimal generators of Lie group elements [29,30].

The design of the AUV motion model, expressed in terms of Lie groups using the Special Euclidean Group, indicates that the instantaneous state of the AUV at any moment *n* is $X_{n}\in SE_{2}(3)=G$, denoted as Equation (1).

(1) $$X_{n}=\left[\begin{array}{ccc} R_{n3\times 3} & v_{n3\times 1} & p_{n3\times 1}\\ 0_{1\times 3} & 1 & 0\\ 0_{1\times 3} & 0 & 1 \end{array}\right]$$

Equation (1) describes the state of AUV on a matrix group over $SE_{2}(3)$, where $R_{n}\in SO(3)$ is the rotation matrix, $v_{n}\in {\mathbb{R}}^{3}$ is the velocity vector of the AUV, and $p_{n}\in {\mathbb{R}}^{3}$ is the position vector of the AUV.

In this system, changes in the AUV’s attitude and position are described using the Lie group $SE_{2}(3)$, and its Lie algebra representation is $se_{2}(3)$, where the element is $\xi \in {\mathbb{R}}^{5}$. The elements in the Lie algebra are utilized to represent the infinitesimal changes in the motion of the AUV. The mapping of the state of the AUV to the Lie algebra, as represented by Equation (2), through the application of transformation $\xi \to \xi^{\wedge }$, is employed to describe the minor fluctuations in the attitude and position of the AUV within the spatial domain. This methodology can be utilized to illustrate the uncertainty of the system.

(2) $$\xi^{\wedge }=\left[\begin{array}{c} \begin{array}{ccc} \xi_{R3\times 3} & \xi_{v3\times 1} & \xi_{p3\times 1} \end{array}\\ 0_{2\times 5} \end{array}\right]$$

where $\xi_{R}$, $\xi_{v}$, and $\xi_{p}$ denote the perturbations of the rotational angular velocity, linear velocity, and position, respectively. These mapping vectors in the Lie algebra are converted into elements in the Lie group, representing continuous or small changes. This is carried out in order to determine the actual position and attitude of the rigid body, which is then used to estimate the system state on the manifold.

### 2.2. IMU Measurement Model

The IMU measurements return the angular velocity and linear acceleration of the AUV itself, while the state changes of the AUV, including the velocity, attitude, and position changes, are obtained by integrating the output of the IMU [31].

The state of the system at moment *n* can be described as $x_{n}=\left[R_{n},v_{n},p_{n},b_{n}\right]$, where the orientation R∈SO(3), velocity $v\in {\mathbb{R}}^{3}$, position $p\in {\mathbb{R}}^{3}$, and offset $b=\left[\begin{array}{cc} b_{\omega } & b_{a} \end{array}\right]\in {\mathbb{R}}^{6}$. The angular velocity and linear acceleration obtained by the IMU in the body coordinate system are used as inputs to the system to describe the state of the AUV itself at moment *n*, as expressed by Equation (3).

(3) $$\left\{ \begin{array}{l} \overset{\cdot }{R(n)}=R(n)\overset{⌢}{\omega }{\left(n\right)}^{\wedge }=R(n){\left(\omega (n)+b_{\omega }(n)+\eta_{\omega }(n)\right)}^{\wedge }\\ \overset{\cdot }{v(n)}=\overset{⌢}{a}(n)=R^{Τ}(n)(a(n)-g)+b_{a}(n)+\eta_{a}(n)\\ \overset{\cdot }{p}=v \end{array}\right.$$

where g is the gravity vector, the control input denoted as $u_{n}={\left[\begin{array}{ccc} \omega_{n} & a_{n} & 0_{1\times 3} \end{array}\right]}^{Τ}$ is set, b denotes the sensor bias, and η∼Ν(0,Q) denotes the observed Gaussian white noise, which represents the motion model of the AUV.

### 2.3. DVL Measurement Model

The DVL employs the Doppler effect of acoustic waves to determine the velocity of the AUV relative to the water bottom or layer of water. The measured value is the velocity of the AUV itself at moment *n*, which is denoted as $v_{n}^{\prime }={\left[\begin{array}{ccc} v_{x} & v_{y} & v_{z} \end{array}\right]}^{Τ}$. Since the velocity measured by the DVL is the combined velocity of the actual velocity of the vehicle in the water and the rotational velocity of the vehicle, the velocity measured by the DVL is calibrated in order to obtain the actual measured value of the AUV linear velocity. For fusion with the IMU, it is necessary to subtract the velocity component $k\times \omega_{n}$ due to the rotation of the vehicle, as expressed in Equation (4), from the velocity measured by the DVL.

(4) $$v_{n}^{\prime }-k\times \omega_{n}={\tilde{v}}_{n}=\left[\begin{array}{ccc} {\tilde{v}}_{x} & {\tilde{v}}_{y} & {\tilde{v}}_{z} \end{array}\right]$$

where ${\tilde{v}}_{n}$ is the actual AUV linear velocity measurement returned by the system at moment *n*, which defines the observation model for DVL as Equation (5)

(5) $$y_{\mathrm{DVL}}=h_{\mathrm{DVL}}(X_{n})+\upsilon_{n}=-R^{Τ}v_{n}+\upsilon_{n}$$

where $h_{\mathrm{DVL}}(X_{n})$ is used to describe the observed function of the DVL, while the estimated linear velocity value of the system at moment *n* is represented by the value of $v_{n}$.$\upsilon_{n}∼Ν(0,R_{n})$ is a Gaussian noise. The AUV linear velocity at moment *n* is transformed to the sensor coordinate system as the sensor measurement at this time to update the velocity state of the AUV.

### 2.4. Depth Sensor Measurement Model

The depth sensor is utilized to measure the vertical position of the AUV in relation to the water surface ${\tilde{z}}_{n}$. This measurement is transformed into the actual position of the AUV on the z-axis at the moment *n* expressed as ${\tilde{p}}_{n}=-R^{Τ}{\left[\begin{array}{ccc} p_{x} & p_{y} & {\tilde{z}}_{k} \end{array}\right]}^{Τ}$, which is utilized to update the state of the system. To fuse the depth information, the depth observation function is transformed to the sensor coordinate system, defining the observation model of the depth sensor as Equation (6).

(6) $$y_{D}=h_{D}(X_{n})+\upsilon_{n}=-R^{Τ}p_{n}+\upsilon_{n}$$

where $h_{D}(X_{n})$ is used to describe the observation function of the depth sensor, while $p_{n}$ is the AUV position information estimated by the system at moment *n*. $\upsilon_{n}∼Ν(0,R_{n})$ is Gaussian noise, and the position component of the observation model on the z-axis is selected as the observation function input to update the model and correct the depth value of the AUV.

### 2.5. Magnetometer Measurement Model

Magnetometers acquire data by measuring the strength and direction of the magnetic field in the surrounding environment. In order to enhance the precision of the orientation during localization, the orientation of the AUV is corrected through the utilization of a magnetometer. The magnetometer measurement is denoted as ${\tilde{m}}_{n}={\left[\begin{array}{ccc} {\tilde{m}}_{x} & {\tilde{m}}_{y} & {\tilde{m}}_{z} \end{array}\right]}^{Τ}$, whereas the canonical magnetic vector at the location where the data were collected is $m={\left[\begin{array}{ccc} m_{x} & m_{y} & m_{z} \end{array}\right]}^{Τ}={\left[\begin{array}{ccc} 0.24494 & -0.002385 & -0.38615 \end{array}\right]}^{Τ}$. The observation model for the magnetometer is defined as Equation (7).

(7) $$y_{M}=h_{M}(X_{n})+\upsilon_{n}=R^{Τ}m_{n}+\upsilon_{n}$$

where $h_{M}(X_{n})$ is used to describe the magnetometer’s observation function, which translates the region’s canonical magnetic vector into the sensor coordinate system as a sensor observation, making the measured direction of travel of the AUV more accurate.

## 3. Multi Sensor Fusion Localization Method

### 3.1. Filter Design

To study the motion of the AUV on a nonlinear manifold, the unscented Kalman filter on the manifold is designed to fuse the information from the IMU, DVL, depth sensors, and magnetometer for 3D attitude estimation of the AUV. The state space function of the AUV, Equation (1), is expressed in the form of a Lie group, which can be described as a discrete-time dynamical system. The predicted state of the AUV at moment *n* is denoted as Equation (8).

(8) $$X_{n+1}=f(X_{n},u_{n},w_{n})$$

where the state $X_{n}$ of the AUV at moment n is on the special Euclidean group $G=SE_{2}(3)$, denoted $X_{n}\in G=\left\{R\in SO(3),v\in {\mathbb{R}}^{3},p\in {\mathbb{R}}^{3}\right\}$, $u_{n}$ is a known input variable, and $w_{n}∼Ν(0,Q_{n})$ is Gaussian white noise.

In Section 2, we described the individual sensor observation models used to observe the state of the AUV, as well as the sensor observation functions utilized by the system. These include the DVL (5), the depth sensor (6), and the magnetometer (7). The fusion of the observation models of these sensors mentioned above into the observation function $y_{n}=h(X_{n},\upsilon_{n})$ can be expressed as Equation (9).

(9) $$y_{n}=h(X_{n},\upsilon_{n})=\left[\begin{array}{c} h_{\mathrm{DVL}}(X_{n})\\ h_{D}(X_{n})\\ h_{M}(X_{n}) \end{array}\right]+\upsilon_{n}=\left[\begin{array}{c} -R^{Τ}v+\upsilon_{n}\\ -R^{Τ}p+\upsilon_{n}\\ R^{Τ}m+\upsilon_{n} \end{array}\right]$$

where $\upsilon_{n}∼Ν(0,R_{n})$ is Gaussian white noise. Based on the information from each observed sensor at each moment, the state mean and covariance at the next moment are predicted and estimated.

### 3.2. Estimating State Uncertainty

The system state model Equation (1) is represented in the form of a Lie group, and in order to represent the estimated state $\tilde{X}$ and the true state X in a uniform mapping, the traditional UKF represents the uncertainty of the system by defining the error $e=X-\tilde{X}$ between the true state and the estimated state. Since the Lie group G is not a vector space and representing the uncertainty of the Lie group by the error may destroy the orthogonality of the rotation matrix when adding noise, the noise in the Lie group cannot be defined in the usual way of attaching noise [28,32]. In this paper, we use the Lie algebra Equation (2) to represent the uncertainty of the system, and realize the mapping relation between Lie groups and Lie algebras by using retraction and inverse retraction functions to ensure that the geometric constraints are strictly adhered to, so that the elements of the Lie groups remain legal after adding noise.

In order to simplify the computational complexity of the unscented Kalman filter, the inter-transformation between the nonlinear manifold and the linear space is realized by designing the retraction function and the inverse retraction function on the filter. The retraction is a smooth mapping from the linear space to the manifold, realizing the local linear approximation on the nonlinear space and mapping the approximation back to the actual position on the manifold. Inverse retraction is the inverse process of remapping, which inversely maps the real state on the manifold back to the linear space, and more simplified operations are realized by these two transformations [33,34].

In order to exponentially map the estimated state and state uncertainty ξ to the new state of the manifold, the retraction function is designed as $X=\varphi (\tilde{X},\xi )\in M$. Likewise, the inverse retraction function is designed as $\xi =\varphi_{\tilde{X}}^{-1}(X)\in {\mathbb{R}}^{d}$ to map the logarithm of the state on the manifold back to the state uncertainty ξ of the estimated state, where φ satisfies φ(X,0)=X. For an AUV whose state function is Equation (1), the retraction function is defined as Equation (10), and the inverse retraction function is defined as Equation (11) to realize the Lie group matrix with Lie algebra uncertainty on the manifold for state estimation and update.

(10) $$\varphi (X,\xi )=\left(\begin{array}{c} \mathrm{Rexp}(\xi_{\left(0:3\right)})\\ v+\xi_{\left(3:6\right)}\\ p+\xi_{\left(6:9\right)} \end{array}\right)$$

(11) $$\varphi_{\tilde{X}}^{-1}(X)=\left(\begin{array}{c} \log(R{\tilde{R}}^{Τ})\\ v-\tilde{v}\\ p-\tilde{p} \end{array}\right)$$

In Equation (10), $R\exp(\xi_{\left(0:3\right)})$ is the exponential mapping of the rotation vector to update the pose, the state uncertainty ξ is a 9-dimensional matrix, and $\xi_{\left(0:3\right)}$ denotes the initial three components of the input ξ representing the components of the rotation. $v+\xi_{\left(3:6\right)}$ represents the update of the current velocity by increment ξ, $\xi_{\left(3:6\right)}$ represents the vector of velocity changes, $p+\xi_{\left(6:9\right)}$ represents the update of the current position by increment ξ, and $\xi_{\left(6:9\right)}$ represents the vector of position changes. Equation (11) inversely differentiates the deviation between a state X on manifold and its estimated state $\tilde{X}$ from the state X itself. The deviation is also the uncertainty of the state ξ. $\log(R{\tilde{R}}^{Τ})$ in Equation (11) is the inverse rotational transformation to obtain the deviation between two poses.

The above UKF designed on manifolds transforms uncertainties in the Lie algebra into state changes in the manifolds by retraction and inverse retraction. When a state function in the form of a Lie group acts on a manifold M, there are two forms of action, the left action and the right action. The left action refers to the mapping Φ: G×M→M of the Lie group G action on the manifold M, expressed as the left multiplication of g and x, where g is Lie group element on the manifold and x is the state at this moment. The left action is written as Φ(g,x), which realizes the transformation of the Lie group on the manifold. Similarly, the right action is another form of transformation, when the Lie group G acts on the manifold M as mapping, Φ: M×G→M, which is expressed as the right multiplication of g and x, where g is Lie group element on the manifold and x is the state at this moment. The right action is written as Φ(x,g). Applying the two transformations to the unscented Kalman filter simplifies the computation of the Lie group by transforming it into the manifold.

When the AUV state X obeys the probability distribution $X∼Ν(\bar{X},P)$, the system’s uncertainty ξ can be shifted using left and right multiplications about the centering of $\bar{X}\in G$. The resulting shifted state X obeys the left-equivariant and right-equivariant Gaussian distributions on G, respectively. For the system Equation (12), with states on $G=SE_{2}(3)$ manifolds, the homogeneous transformation matrix Τ is computed from the elements of the Lie algebra Equation (2), and then the uncertainty of the states is computed using the homogeneous transformation matrix Equation (13).

(12) $$SE_{2}(3)=\left\{Τ=\left[\begin{array}{ccc} \begin{array}{l} R\\ 0^{Τ} \end{array} & \begin{array}{l} v \end{array} & \begin{array}{l} p\\ Ι \end{array} \end{array}\right]\in {\mathbb{R}}^{5\times 5}|R\in SO(3),v\in {\mathbb{R}}^{3},p\in {\mathbb{R}}^{3}\right\}$$

(13) $$Τ(\xi )=\exp(\xi^{\wedge })=\left[\begin{array}{ccc} \begin{array}{l} \exp(\xi_{R})\\ 0^{Τ} \end{array} & \begin{array}{l} J\xi_{v} \end{array} & \begin{array}{l} J\xi_{p}\\ Ι \end{array} \end{array}\right]$$

Using the homogeneous transformation matrix, the left-equivariant retraction function Equation (14) is designed for the state of the AUV in the system using the left multiplication of the Lie group for state updating. Likewise, the right-equivariant retraction function Equation (16) is designed for state updating using the right multiplication of the Lie group. Equations (14) and (16), respectively, correspond to inverse retraction function Equations (15) and (17) to reflect the uncertainty of the system.

(14) $$left\;\varphi (X,\xi )=\left(\begin{array}{c} RΤ{\left(\xi \right)}_{R}\\ v+RΤ{\left(\xi \right)}_{v}\\ p+RΤ{\left(\xi \right)}_{p} \end{array}\right)$$

(15) $$left\;\varphi_{\tilde{X}}^{-1}(X)=\log(X^{-1}\tilde{X})=\left(\begin{array}{c} \ln{\left(R^{\prime }\right)}^{\vee }\\ J^{-1}v^{\prime }\\ J^{-1}p^{\prime } \end{array}\right)$$

(16) $$right\;\varphi (X,\xi )=\left(\begin{array}{c} Τ{\left(\xi \right)}_{R}R\\ Τ{\left(\xi \right)}_{R}v+Τ{\left(\xi \right)}_{v}\\ Τ{\left(\xi \right)}_{R}p+Τ{\left(\xi \right)}_{p} \end{array}\right)$$

(17) $$right\;\varphi_{\tilde{X}}^{-1}(X)=\log({\tilde{X}}^{-1}X)=\left(\begin{array}{c} \ln{\left(R^{\prime \prime }\right)}^{\vee }\\ J^{-1}v^{\prime \prime }\\ J^{-1}p^{\prime \prime } \end{array}\right)$$

where ${\left(\cdot \right)}^{\prime }$ denotes the state resulting from state multiplication ${\tilde{X}}^{-1}X$, ${\left(\cdot \right)}^{\prime \prime }$ denotes the state resulting from state multiplication $X^{-1}\tilde{X}$, and *J* is the Jacobian matrix associated with the rotation. The use of the left-equivariant and right-equivariant retraction functions with the inverse retraction function allows for efficient handling of states and uncertainties on manifolds, ensuring that the system maintains geometrical constraints and computational simplicity during updating, and realizing 3D attitude estimation of the AUV.

### 3.3. Predicting System Status

The algorithm of the proposed multi-sensor fusion localization method designed based on unscented Kalman filtering on manifolds is displayed in Algorithm 1, which includes two steps.

(1) The prediction step in lines 3–7 of the algorithm: update the system’s state and covariance by propagating the IMU measurements.

(2) The updating step in lines 8–15 of the algorithm: further correct the state of the system by fusing the observations from the DVL, the depth sensors, and the gyroscope.
**Algorithm 1:** A Multi-sensor Fusion Underwater Localization Method**1 Input**: Angular velocity ω, Acceleration a     DVL, Depth Sensor and Magnetometer measurement     Observing noise $R_{n}$, System noise $Q_{n}$, State input noise $w_{n}$, Sigma point α 
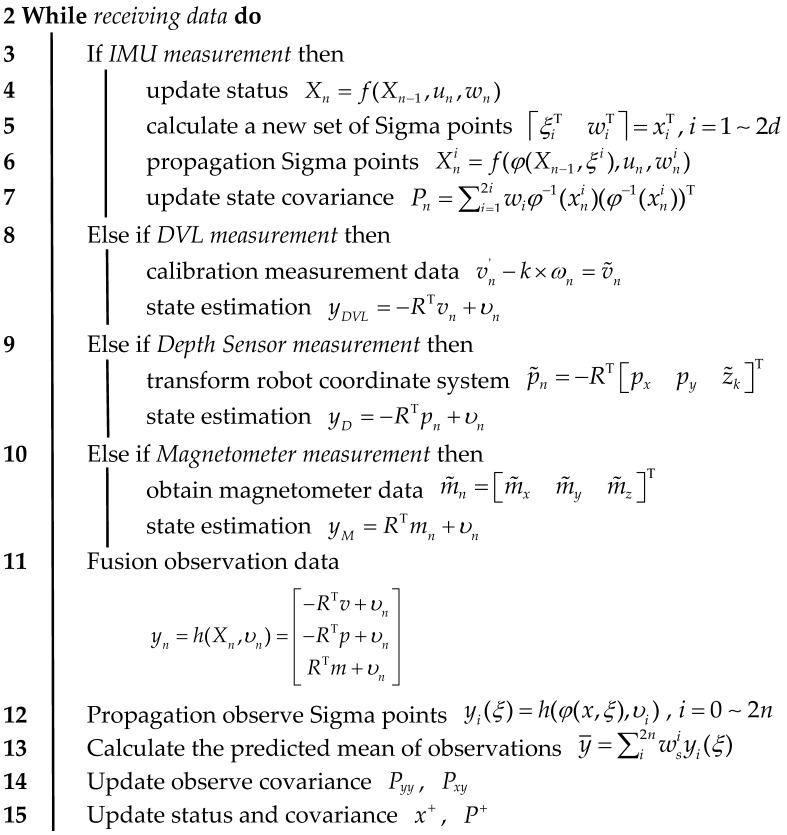
**16 End**

The two steps are described in detail as the following:

(1) Propagation Step.

During the propagation process, in order to avoid linearizing the system, some Sigma points around the current state estimate and covariance matrix are selected by the unscented Kalman filter on the manifolds to approximate the state distribution, and then these points are propagated through the nonlinear function Equation (3) to obtain the new state distribution. Considering the noise $w_{n}$ in the state model Equation (8), the covariance matrix of the state x at each moment is augmented and denoted as $P_{aug}=diag(P,R)$ and the mean of the state x is re-expressed as $\bar{x}={\left[\begin{array}{cc} 0^{Τ} & {\bar{w}}^{Τ} \end{array}\right]}^{Τ}$. At this point, the state X of the system is an *n*-dimensional state vector with a mean of $\bar{x}$ and a variance of $P_{aug}$. Constructing a Sigma point by unscented transform (UT) is expressed as Equations (18) and (19).

(18) $$x_{i}=\bar{x}+col{\left(\sqrt{(\lambda +d)P_{aug}}\right)}_{i}\;,\;i=1∼d$$

(19) $$x_{i}=\bar{x}-col{\left(\sqrt{(\lambda +d)P_{aug}}\right)}_{i}\;,\;i=d+1∼2d$$

where λ is the scale factor. The larger λ is, the further away the sigma point is from the mean value of the state; the smaller λ is, the closer the sigma point is to the mean value of the state. ${\left(\sqrt{(\lambda +d)P_{aug}}\right)}_{i}$ denotes the *i*-th column of the matrix square root $\sqrt{(\lambda +d)P_{aug}}$. The corresponding weights of the sampling points are calculated by Equation (20).

(20) $$\left\{ \begin{array}{l} w_{m}^{\left(0\right)}=\frac{\lambda }{d+\lambda }\\ w_{c}^{\left(0\right)}=\frac{\lambda }{d+\lambda }+(1-\alpha^{2}+\beta )\\ w_{m}^{\left(i\right)}=w_{c}^{\left(i\right)}=\frac{\lambda }{2(d+\lambda )}\;,\;i=1∼2d \end{array}\right.$$

When x follows a Gaussian distribution, β=2 is the optimal parameter. By setting the input state x’s systematic uncertainty ξ∼Ν(0,P), the systematic uncertainty is added as noise to the set of Sigma points at this point, and mapped to the new set of Sigma points by Equation (21).

(21) $$\left[\begin{array}{cc} \xi_{i}^{Τ} & w_{i}^{Τ} \end{array}\right]=x_{i}^{Τ}$$

In order to enact the Lie group on the manifold, the three action methods designed in the previous section, retraction, left-equivariant retraction, and right-equivariant retraction, are selected. The designed retraction function and inverse retraction function are used to propagate the Sigma points and compute the new state mean and covariance based on the propagated Sigma points on the tangent space of the manifold. The results are mapped back to the manifold to obtain the new estimate and uncertainty of the state after the time update.

(2) Update Step.

First, Sigma points are generated based on the new state estimates obtained from the propagation process and their covariance matrices. The Sigma points at this point not only capture the mean and variance, but also retain the higher-order statistical properties of the original probability distribution. The new set of Sigma points is propagated through the observation model Equation (22). Each Sigma point on the manifold is transformed into the expected measurement space by means of the designed retraction function and inverse retraction function to obtain the expected measurement results.

(22) $$y_{i}(\xi )=h(\varphi (x,\xi ),\upsilon_{i})\;,\;i=0∼2n$$

The mean in Equation (23) and covariance in Equation (24) of the measurements were predicted after performing a weighted Equation (20) on the new set of Sigma points.

(23) $$\bar{y}=\sum_{i=0}^{2n}w_{s}^{i}y_{i}(\xi )$$

(24) $$P_{yy}=\sum_{i=0}^{2n}w_{c}^{i}(y_{i}(\xi )-\bar{y}){\left(y_{i}(\xi )-\bar{y}\right)}^{Τ}$$

Subsequently, the nonlinear observations of the Sigma point set state measurements are calculated and the Kalman gain is computed using the covariance of the predicted measurements in Equation (24) and the cross-covariance in Equation (25). This Kalman gain will be used to determine the degree of influence of the difference between the actual and predicted measurements.

(25) $$P_{xy}=\sum_{i=0}^{2n}w_{c}^{i}(x_{i}-\bar{x}){\left(y_{i}-\bar{y}\right)}^{Τ}$$

(26) $$\bar{\xi }=K(y-\bar{y})$$

where $K=P_{xy}P_{yy}^{-1}$ denotes the Kalman gain of the system, and finally, the Kalman gain and measurement residuals are used to update the system’s state estimate, Equation (27), with the covariance, Equation (28).

(27) $$x^{+}=\varphi (\bar{x},\bar{\xi })$$

(28) $$P^{+}=P-P_{xy}{\left(P_{xy}P_{yy}^{-1}\right)}^{Τ}$$

Since the system is carried out on the manifold, the updated state needs to be mapped back to the manifold again, otherwise the linear combination of results may cause the results to deviate from the manifold. At this point, the system fuses the information from multiple sensors to complete the state prediction and update, and $x^{+}$ and $P^{+}$ denote the final state estimation result and covariance of the system, respectively.

## 4. Experimental Result and Analysis

### 4.1. Underwater Cave Dataset

**Figure 2:**
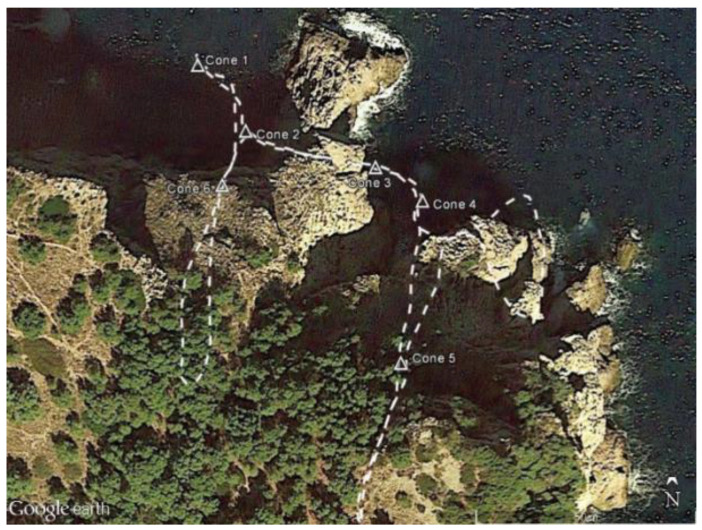
External environment of underwater caves.

To study the localization effect of the method proposed in this article, the method is performed on a dataset collected by an AUV named Sparus II. The collection of the dataset was carried out by Mallios et al. [35] from an unstructured natural underwater cave in the L’Escala area of the Costa Brava, Spain. Figure 2 shows the external view of the cave with the route of the collection trajectory (white dotted line). The data were collected with the setup of six fixed cones, and the position of each cone is also labeled cones 1–6 in Figure 2. A diver led the AUV through the cave and the shuttle route passed through each cone twice. The location of the cones acts as the reference to verify the accuracy of the localization method [36].

**Figure 3:**
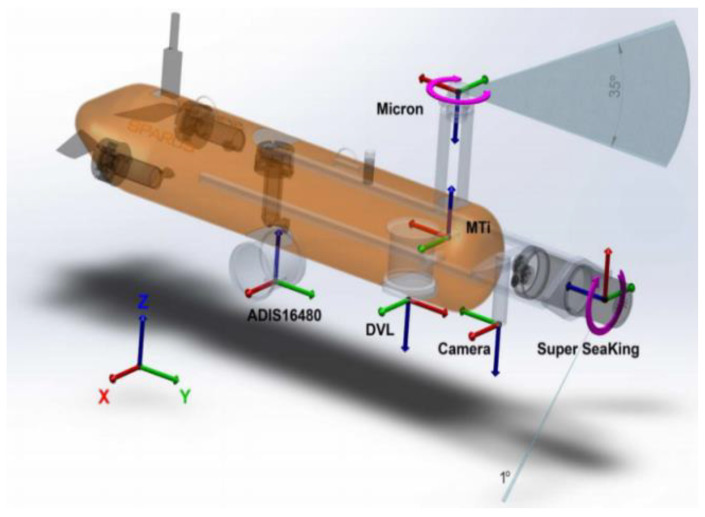
Sparus II AUV internal structure.

Figure 3 shows the internals of the Sparus II AUV, which is equipped with a variety of sensors such as IMU, DVL, gyroscope, and depth sensor. The data were collected while the AUV was manually guided through the cave. Table 1 shows the system’s main on-board sensors.

### 4.2. Experimental Effect of Fusion Algorithm

Using the individual sensor data returned by the AUV in the underwater cave, the above proposed unscented Kalman filter fusion method based on manifolds is applied to estimate the 3D attitude of the AUV. The parameters of each sensor are shown in Table 1. The AUV is localized by fusing the IMU, DVL, and depth sensor information with the above-designed conventional manifolds UKF, left-equivariant UKF, and right-equivariant UKF, respectively.

**Figure 4:**
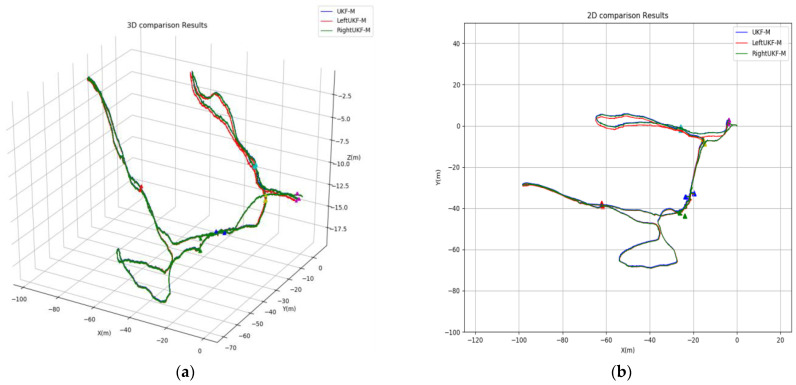
Underwater cave trajectory estimation results for the three UKF-M filters proposed in this paper. (**a**) The 3D trajectory estimation results; (**b**) 2D trajectory estimation results.

In Figure 4, we plot the results of the comparison of the three localization methods of the conventional manifolds UKF-M (blue line), the left-equivariant UKF-M (red line), and the right-equivariant UKF-M (green line) among the three filters designed in this paper. Different colored triangles are used to represent the positions of the six cones in the trajectory estimation. The three-axis coordinate system represents the position of the AUV in meters. It can be seen from the figure that the trajectory estimates obtained from the three methods are very similar.

**Figure 5:**
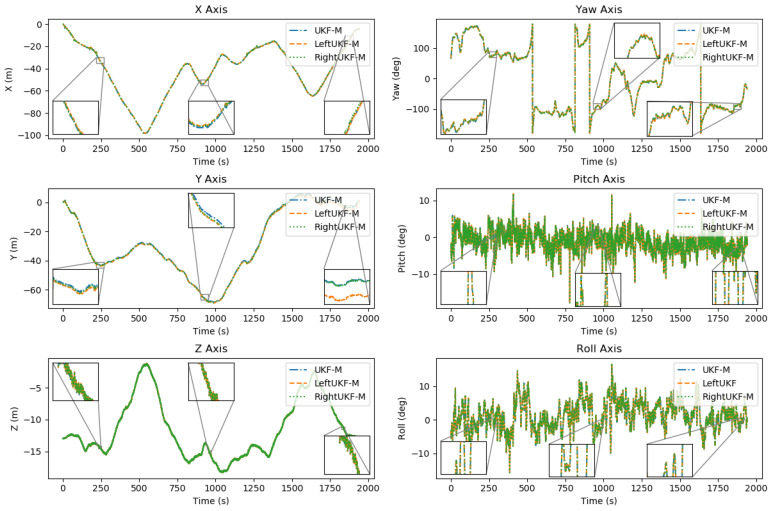
Transformations of the three filters in the three positional coordinate axes x, y, and z with the three attitude angles of yaw, pitch, and roll axes over time.

In order to better observe the difference between the trajectories obtained by the three methods, the transformations of the three filters in the three position coordinate axes x, y, z and the three attitude angles of the yaw, pitch, and roll axes with respect to time are displayed in Figure 5. Three regions in the pre and post time periods were randomly selected for the zoomed-in display. The estimation of the position coordinate axes and the attitude angles of the three filters, UKF-M, LeftUKF-M, and RightUKF-M, are somewhat consistent, which shows that these filters have a similar performance in processing these data. Moreover, the three filters have significant variations in the yaw, pitch, and roll axes (especially in the pitch and roll axes), which show a better dynamic response capability. The three filters perform well in processing different coordinates and attitude angles without significant degradation or deviation, showing their validity and reliability.

Due to the consistency of the three filters UKF-M, LeftUKF-M, and RightUKF-M, one of them, i.e., the LeftUKF-M, is selected for comparison for the sake of the convenience of the result evaluation. Three other commonly used navigation and localization methods, EKF, RI-EKF, and Dead Reckoning (DR), are chosen as the reference of comparison to evaluate the performance of the proposed methods. Among them, EKF has been widely used in localization studies of nonlinear systems for decades, a right-invariant extended Kalman filter (RIEKF) brought out by Easton R. Potokar’s team [24] was applied to underwater localization on Lie groups, while DR has been used as one of the most basic and direct localization methods. These three methods can be representative of the regular navigation and localization performance.

**Figure 6:**
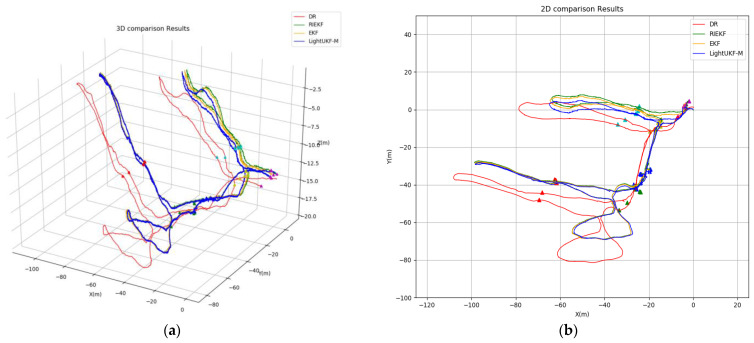
Underwater cave trajectory estimation results of DR, RIEKF, EKF, and LeftUKF-M proposed in this paper. (**a**) The 3D trajectory estimation results; (**b**) 2D trajectory estimation results.

**Figure 7:**
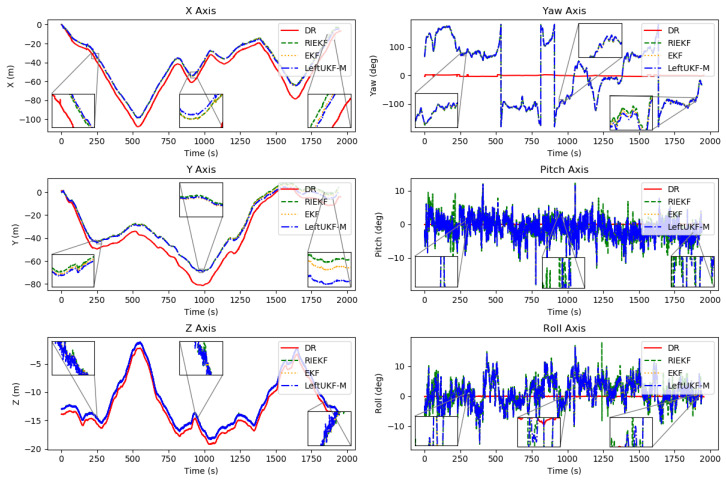
Transformation of DR, RIEKF, EKF, and LeftUKF-M in x, y, and z axes and yaw, pitch, and roll axes with time.

In Figure 6, we plot the trajectories estimated by the LeftUKF-M (blue line), DR (red line), EKF (orange line), and RIEKF (green line). The cones are still represented by different colored triangles. It can be seen that both the proposed methods, EKF and RIEKF, are able to correct the cumulative error in trajectory estimation. Similar to Figure 5, Figure 7 shows the transformation of DR, RIEKF, EKF, and LeftUKF-M in the x, y, and z axes and yaw, pitch, and roll axes over time. The estimation results on the position coordinates of the x, y, and z axes show that the three filters, DR, RIEKF, and LeftUKF-M, have comparable performances in position estimation with some consistency. In the attitude change plots, DR shows significant inaccuracy as the yaw, pitch, and roll remain almost constant throughout the process, while RIEKF, EKF, and LeftUKF-M show more significant changes in the yaw, pitch, and roll axes, showing better dynamic responsiveness. Overall, RIEKF, EKF, and LeftUKF-M perform more consistently and stably in the estimation of each coordinate axis and pose angle, and the DR filter performs poorly in the pose estimation.

Since it is not possible to use GPS to measure the ground truth in underwater environments, in order to quantitatively rate the accuracy of the proposed methods, we use the six cones in the dataset to rate the results of these six trajectory estimates.

Two criteria were used to quantitatively assess the accuracy of localization using the filter. The first criterion: (1) the AUV trajectory passes through the six cones successively and the same cone is passed through twice, and the position estimation error of passing through the same cone successively is quantitatively analyzed. The position error of the AUV passing through the same cone twice is shown in Table 2.

The second criterion: (2) the placement of each cone is fixed and known, and the AUV passes through six cones according to the path 1→2→3→4→5→5→4→3→2→6→6→1. The distances between two neighboring cones in the estimated trajectory of the filter are compared with the actual distances of the ground. The distances between neighboring cones that the AUV passes through according to the paths of the proposed methods are demonstrated in Table 3. The absolute value of the error between each method and the ground truth distance are displayed in Table 4.

In the above two criterion, the optimal estimates are marked in bold, and in Table 2, the three proposed filters show a clear advantage over RIEKF, EKF, and DR in the positional error of the AUV passing through the same cone twice. In Table 3 and Table 4, the three proposed filters, EKF and RIEKF, show better performances in the distance error of neighboring cones in accordance to the ground truth, whereas the difference between DR and the ground truth is larger. In conclusion, through the above analysis, the proposed method has a clear advantage in the first criterion, and in the second criterion, the results are slightly better than RIEKF. Hence, the proposed method shows obvious advantages in the correction of the cumulative error in the trajectory estimation and in the improvement of the accuracy of localization.

## 5. Conclusions

In this paper, we derive a multi-sensor fusion localization method that can be applied to underwater navigation and localization using a recently developed unscented Kalman filter on manifold framework for underwater vehicle systems. Information from common underwater sensors, the IMU, DVL, depth sensor, and magnetometer, are used to correct the state and position of the AUV during navigation in order to eliminate the inaccuracy of localization due to accumulated errors during navigation. By fusing the underwater depth sensor information, the 3D trajectory estimation of the AUV is realized, and the proposed method is validated using an underwater cave dataset. The experiments demonstrate that the proposed method can be applied to underwater environments and can effectively improve the accuracy of underwater localization. A comparison with EKF and RIEKF based on Lie groups reveals that the localization accuracy has been improved. Future work will be integrating more underwater sensors, such as a sonar and binocular camera, and further improving the localization accuracy based on this method. And furthermore, the mapping of the underwater environment can be realized based on the results of sensor integration and the improved localization.

## Figures and Tables

**Table 1:** Sensors on board Sparus II AUV.

Sensors	Specifications	
AHRS—XSens MTi	Angular resolution	0.05 deg
	Repeatability	0.2 deg
	Static accuracy (roll/pitch)	0.5 deg
	Static accuracy (Heading)	1 deg
	Dynamic accuracy	2 deg RMS
DVL—LinkQuest NavQuest 600 Micro	Frequency	600 kHz
	Velocity accuracy	0.2% ± 1 mm/s
	Altitude	0.3–140 m
	Max ping rate	5 Hz
Depth—DS2806 HPS-A	Pressure range	0–5 bar
	Output span	4 V ± 1%
	Output zero	1 V ± 1% of span
	Repeatability	±0.25% of span
Profiling sonar—Tritech Super SeaKing DFP	Frequency	0.6|1.1 MHz
	Max range	80|40 m
	Beamwidth	2|1 deg
	Scan rate (360 deg sector)	4–25 s
Down-looking analog camera	System	PAL
	Resolution	384 × 288 pixels
	Lighting source	2 × 24 W HID

**Table 2:** Position error of AUV passing through the same cone twice.

Cone Num	DR	UKF-M	LeftUKF-M	RightUKF-M	EKF	RIEKF
1	6.60	2.76	**2.11**	2.52	2.66	3.74
2	3.84	2.21	2.02	**1.97**	2.30	2.96
3	2.81	2.41	**1.92**	2.14	2.89	3.37
4	3.54	2.99	2.98	**2.84**	3.48	3.39
5	2.44	0.99	**0.97**	0.98	1.00	1.03
6	4.37	1.72	**1.36**	1.67	2.29	1.87
Avg.	3.93	2.18	**1.89**	2.02	2.44	2.73

**Table 3:** The distance between AUVs passing through cones in sequence.

Cone Distance	Ground Truth	DR	UKF-M	LeftUKF-M	RightUKF-M	EKF	RIEKF
1→2	19	17.07	17.51	17.50	17.44	17.42	17.82
2→3	32	31.53	31.32	31.36	31.28	30.81	30.88
3→2	32	31.38	32.29	32.13	32.11	31.65	31.96
3→4	16	12.52	14.35	14.46	14.10	14.19	14.11
4→3	16	13.23	17.79	17.68	17.66	17.71	17.77
6→1	30	26.42	31.50	31.46	31.80	30.96	31.03

**Table 4:** Absolute value of error between the true distance from the ground.

Cone Distance	DR	UKF-M	LeftUKF-M	RightUKF-M	EKF	RIEKF
1→2	1.93	1.49	1.50	1.56	1.58	**1.18**
2→3	**0.47**	0.68	0.64	0.72	1.19	1.12
3→2	0.62	0.29	0.13	0.11	0.35	**0.04**
3→4	3.48	1.65	**1.54**	1.90	1.81	1.89
4→3	2.77	1.79	**1.68**	1.66	1.71	1.77
6→1	3.58	1.50	1.46	1.80	**0.96**	1.03
Avg.	2.14	1.23	**1.16**	1.29	1.27	1.17

## Data Availability

The underwater cave dataset used in this paper is available at https://cirs.udg.edu/caves-dataset/ (accessed on 17 August 2024).
